# Supplementary material for: Macrophages disseminate pathogen associated molecular patterns through the direct extracellular release of the soluble content of their phagolysosomes
Source: Nat Commun. 2022 Jun 2;13:3072. doi: 10.1038/s41467-022-30654-4 (PMC9163141; doi:10.1038/s41467-022-30654-4)
Supplement: Supplementary file 1 — Supplementary Information [file 41467_2022_30654_MOESM1_ESM.pdf]

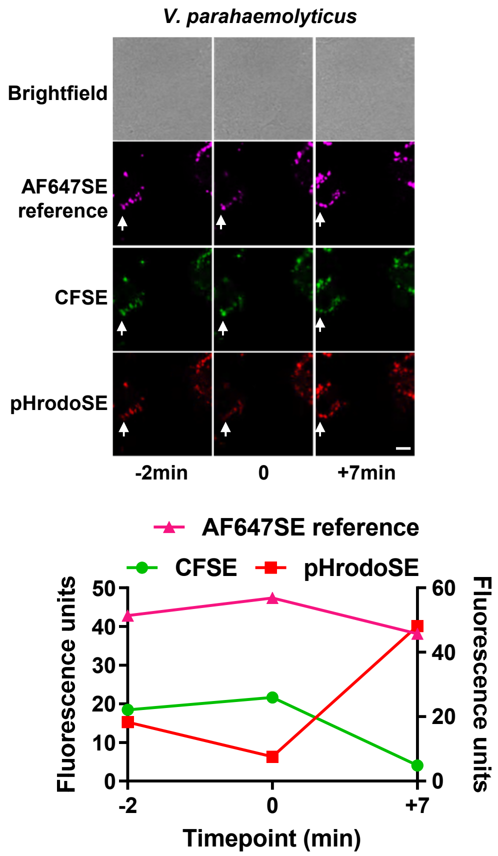

V. parahaemolyticus
Brightfield
AF647SE reference
CFSE
pHrodoSE
-2min
0
+7min
AF647SE reference
CFSE
pHrodoSE
Fluorescence units
50
40
30
20
10
0
60
40
20
0
-2
0
+7
Timepoint (min)

Supplementary Fig. 1. Transient neutralization of phagolysosomal lumenal pH after phagocytosis of the Gram-positive *V. parahaemolyticus*. Representative sequential images of BMMØs and corresponding trace diagram following the phagocytosis of paraformaldehyde-fixed *V. parahaemolyticus* labelled with the pH-sensitive fluors CFSE (green) and pHrodoSE (red), and the reference fluorophore AF647SE (magenta). Transient neutralization of phagolysosomal lumenal pH was demonstrated by the transient neutralization of phagolysosomes, as evidenced by the concomitant increase in CFSE fluorescence (green) and loss of pHrodoSE fluorescence (red), followed by re-acidification of the phagolysosomal lumen. AF647SE reference is plotted on the right axis. CFSE and pHRodoSE are plotted on the left axis. Images were captured using a Leica SP5 confocal microscope between 45 min and 3 h post-phagocytosis at 37°C. Time 0 represents an eructophagy event. Scale bar denotes 5 µm.

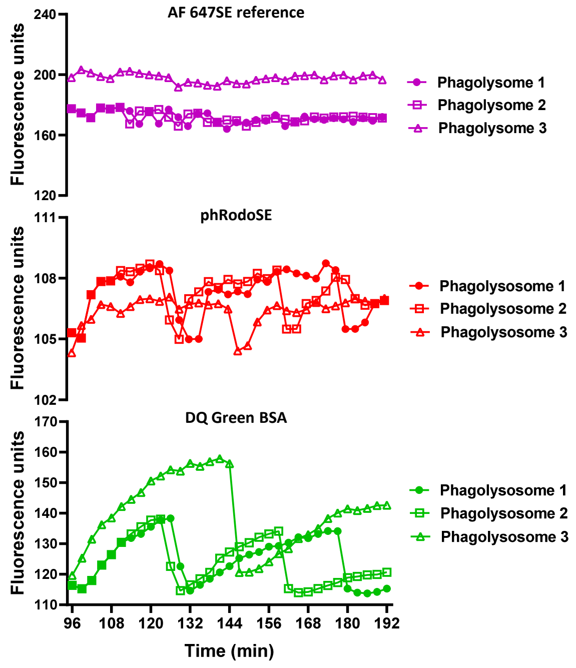

AF 647SE reference
Fluorescence units
240
200
160
120
Phagolysome 1
Phagolysome 2
Phagolysome 3
phRodoSE
111
108
105
102
Phagolysosome 1
Phagolysosome 2
Phagolysosome 3
DQ Green BSA
170
160
150
140
130
120
110
96
108
120
132
144
156
168
180
192
Time (min)

Supplementary Fig. 2. Certain phagolysosomes undergo multiple digestion-release iterations. Representative trace diagrams of phagolysosomes containing reporter particles labelled with DQ Green BSA (green), pHrodoSE (red) and the reference fluorophore AF647SE (magenta). Eructophagy was demonstrated by the simultaneous loss of soluble peptide products of DQ Green BSA (green) and neutralization of the phagolysosome (red), followed by resumption of proteolysis and re-acidification. BMMØs were imaged using an IN Cell Analyzer 2000 between 90 min and 3 h post-phagocytosis at 37°C. Single phagosome tracking and fluorescent quantification were carried out using the IN Cell Investigator software (version 1.8.3) and visualized with Spotfire graphical software.

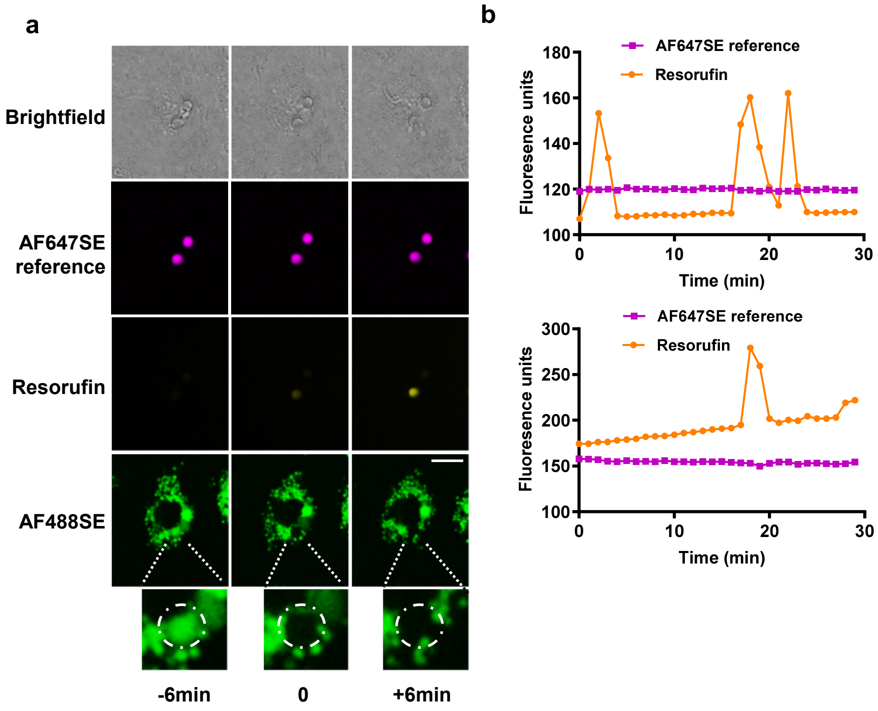

a
Brightfield
AF647SE reference
Resorufin
AF488SE
-6min
0
+6min
b
AF647SE reference
Resorufin
Fluoresence units
Time (min)
180
160
140
120
100
0
10
20
30
AF647SE reference
Resorufin
Fluoresence units
Time (min)
300
250
200
150
100
0
10
20
30

Supplementary Fig. 3. Eructophagy is the transitory bi-directional exchange of soluble material between phagolysosomes and the extracellular environment. (a) BMMØs were pulsed overnight with AF488-labeled dextran (150kDa) and chased to lysosomes over 2 h prior to the phagocytosis of cellulase-coupled reporter particles labeled with AF647SE. The membrane-impermeant substrate, resorufin cellobioside, was added to assay medium 30 min post-phagocytosis. Sequential confocal images depict a phagolysosome containing AF488-labeled dextran and particle-restricted cellulase undergoing eructophagy. Immediately following the loss of AF488-labeled dextran (green) from the phagolysosome at T 0, hydrolysis of the extracellular cellulase substrate (yellow) was detected within the phagolysosome. Time 0 represents an eructophagy event. Scale bars denote 10 µm. (b) Trace diagrams of phagolysosomes of a single phagolysosome following the phagocytosis of 3.0 µm silica particles conjugated to cellulase and the reference fluorophore AF647SE. Eructophagy was demonstrated by the transient fluorescent signal generated by direct hydrolysis of the membrane impermeable resorufin cellobioside within the phagolysosome by particle-restricted cellulase. Resorufin cellobioside was added to the assay medium immediately prior to imaging. Images were captured using an IN Cell Analyzer 2000 between 90 min and 3 h post-phagocytosis at 37°C. Single phagosome tracking and fluorescent quantification were carried out using the IN Cell Investigator software (version 1.8.3) and visualized with Spotfire graphical software. Time 0 represents the beginning of imaging after substrate addition.

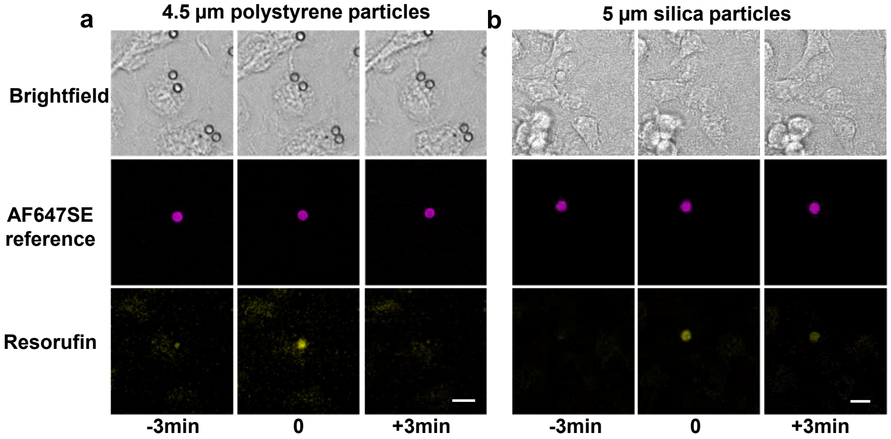

a
4.5 µm polystyrene particles
b
5 µm silica particles
Brightfield
AF647SE
reference
Resorufin
-3min
0
+3min
-3min
0
+3min

Supplementary Fig. 4. Eructophagy is not limited by size or material of phagocytosed particles as phagolysosomes containing large silica or polystyrene reporter particles undergo eructophagy. Representative sequential images of BMMØs following the phagocytosis of **(a)** 4.5 µm polystyrene particles or **(b)** 5.0 µm silica particles conjugated to α-amylase and the reference fluorophore AF647SE. Eructophagy was demonstrated by the transient fluorescent signal generated by direct hydrolysis of the membrane impermeable resorufin maltotriose (yellow) within the phagolysosome by particle-restricted α-amylase. Resorufin maltotriose was added to the assay medium 30 min post-phagocytosis. Images were captured using **(a)** an IN Cell Analyzer 2000; or **(b)** a Leica SP5 confocal microscope between 90 min and 3 h post-phagocytosis at 37°C. Time 0 represents an eructophagy event. Scale bars denote 10 µm.

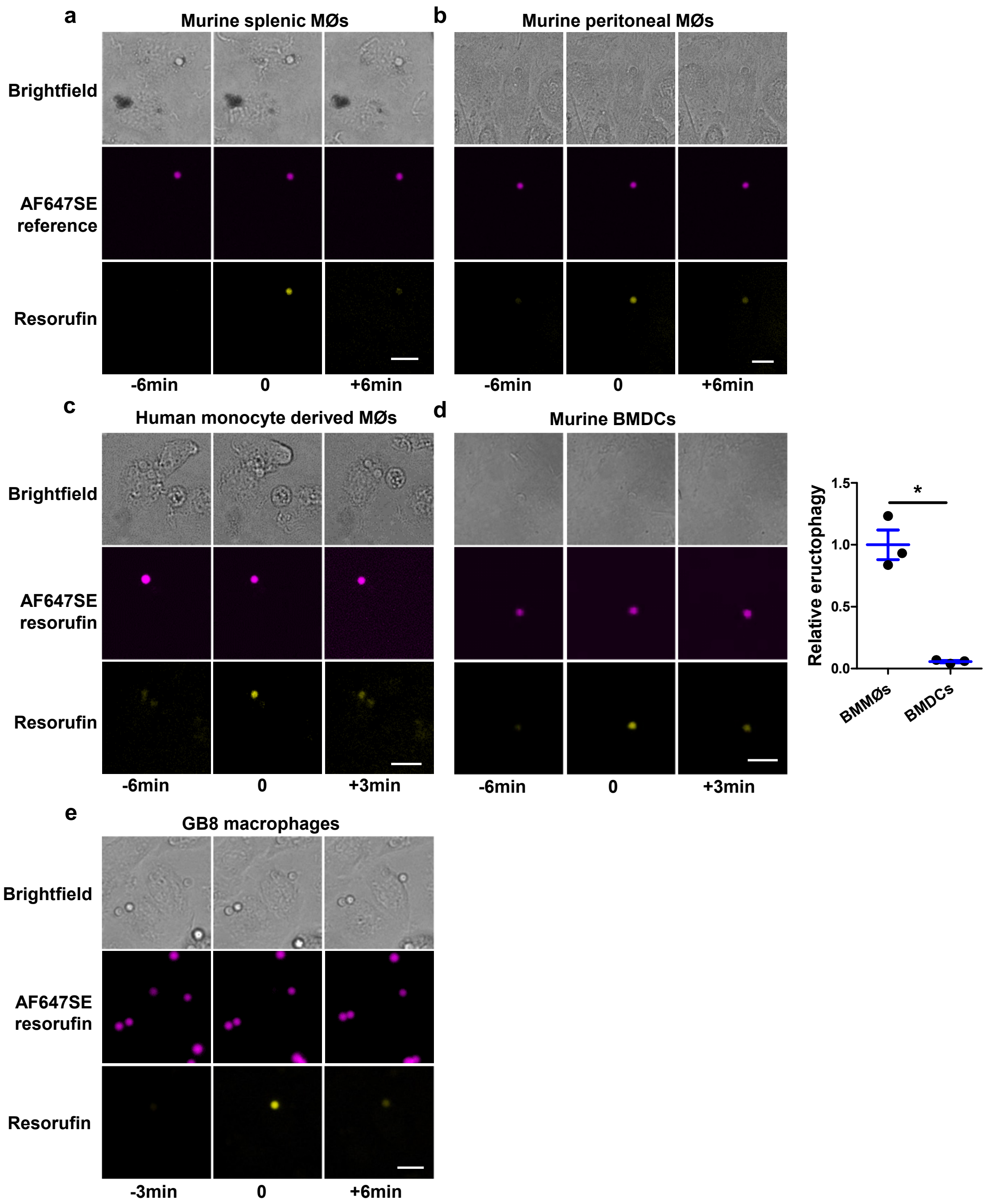

a
Murine splenic MØs
Brightfield
AF647SE reference
Resorufin
-6min
0
+6min
b
Murine peritoneal MØs
-6min
0
+6min
c
Human monocyte derived MØs
Brightfield
AF647SE resorufin
Resorufin
-6min
0
+3min
d
Murine BMDCs
-6min
0
+3min
Relative eructophagy
1.5
1.0
0.5
0.0
*
BMMØs
BMDCs
e
GB8 macrophages
Brightfield
AF647SE resorufin
Resorufin
-3min
0
+6min

Supplementary Fig. 5. Eructophagy occurs in several primary macrophage and dendritic cell types. Representative sequential images of **(a)** CD11b$^+$ macrophages isolated from murine spleens, **(b)** murine peritoneal macrophages, **(c)** human monocyte-derived macrophages, **(d)** murine bone marrow-derived dendritic cells (BMDC) and, **(e)** GB8 macrophages (see text for details). Eructophagy was demonstrated by the transient fluorescent signal generated by direct hydrolysis of the extracellular substrate resorufin maltotriose (yellow) within the phagolysosome by particle-restricted α-amylase. The membrane-impermeant α-amylase substrate resorufin maltotriose was added to assay medium 30 min post-phagocytosis of reporter particles. Images were captured on an IN Cell Analyzer 2000 for 2 h, 90 min post-phagocytosis. Time 0 represents an eructophagy event. Scale bars denote 10 µm. (d) n=3 biologically independent experiments. Error bars presented as means ± SEM. *P = 0.0014 by Student's two-tailed unpaired *t*-test.

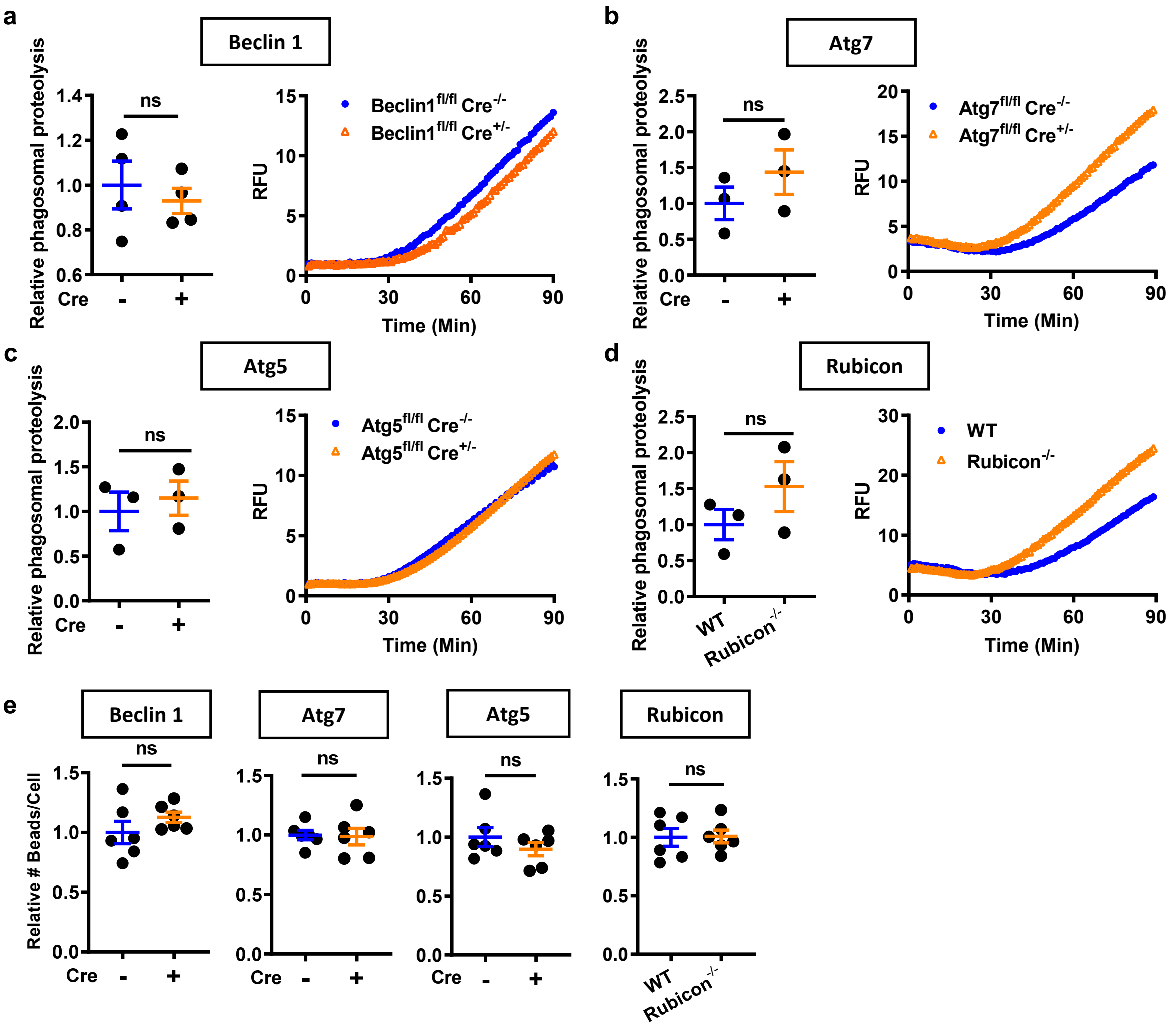

a
Beclin 1
Relative phagosomal proteolysis
ns
Cre - +
RFU
Beclin1fl/fl Cre-/-
Beclin1fl/fl Cre+/-
Time (Min)
b
Atg7
Atg7fl/fl Cre-/-
Atg7fl/fl Cre+/-
c
Atg5
Atg5fl/fl Cre-/-
Atg5fl/fl Cre+/-
d
Rubicon
WT
Rubicon-/-
e
Relative # Beads/Cell

Supplementary Fig. 6. Phagolysosomal kinetics in BMMØs deficient in key autophagy genes. Bulk proteolytic activity in phagosomes and bead uptake by macrophages were evaluated following phagocytosis of fluorescently-labelled, IgG-coupled experimental particles. **(a-d)** Mean slopes and trace diagrams for intraphagosomal bulk proteolysis activity in BMMØs conditionally deficient in **(a)** Beclin1, **(b)** ATG7, **(c)** ATG5 or deficient in **(d)** Rubicon and their corresponding WT controls were evaluated immediately following phagocytosis of IgG-coupled experimental particles labelled with DQ green-BSA. Proteolytic activity was measured in the FLUOstar® Optima by the fluorescence liberated through proteolysis of particle-associated DQ green-BSA relative to the calibration fluorescence, AF594SE. n = 3. **(e)** Relative bead uptake in BMMØs deficient for the indicated genes and their corresponding WT controls after phagocytosis of silica beads labelled with Oregon Green. 1 hour of post-phagocytosis, cells were immediately washed and imaged with the IN Cell Analyzer 2000. The quantity of phagocytosed beads per phagosome was manually numerated with Image J. (a) n=4, (b-d) n=3 biologically independent experiments. (e) n=3, 2 FOV over 3 independent experiments. Error bars presented as means ± SEM. P = (a) 0.58 (b) 0.32 (c) 0.63 (d) 0.26 (e) 0.25, 0.88, 0.32 and 0.93 by Student's two-tailed unpaired t-test.

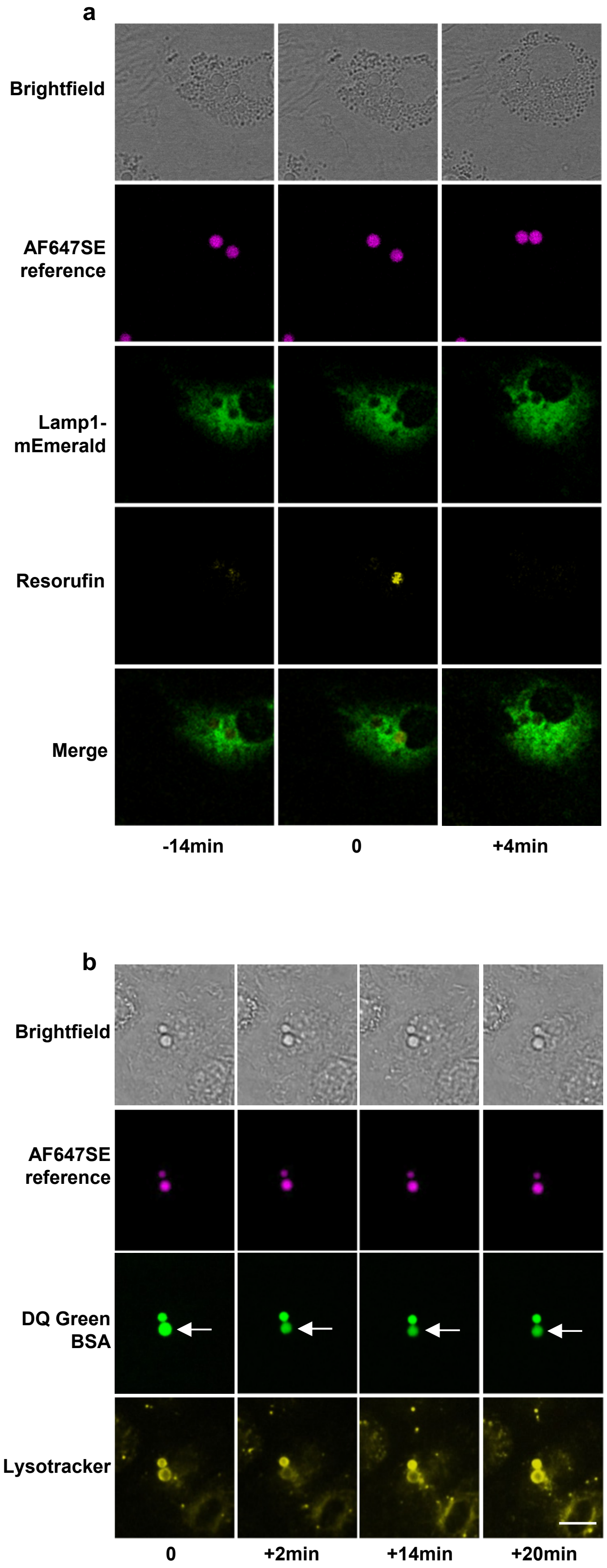
a
Brightfield
AF647SE reference
Lamp1-mEmerald
Resorufin
Merge
-14min
0
+4min
b
Brightfield
AF647SE reference
DQ Green BSA
Lysotracker
0
+2min
+14min
+20min

Supplementary Fig 7. Phagolysosomal characteristics before, during and after eructophagy. (a) Phagolysosomes retain the lysosomal marker LAMP1 during eructophagy. Representative images of a phagolysosome undergoing eructophagy (yellow/magenta) in a BMMØ expressing mEmerald-tagged rat LAMP1 (green). Eructophagy was detected by the transient fluorescent signal generated by direct hydrolysis of the membrane impermeant substrate resorufin cellobioside (yellow) within the phagolysosome by particle-restricted cellulase. Images were captured using a Leica SP5 confocal microscope between 90 min and 2 h post-phagocytosis at 37°C. **(b)** Phagolysosomes retain the ability to fuse with lysosomes immediately following eructophagy. Representative sequential images of BMMØs after phagocytosis of 3.0 µm DQ Green BSA-conjugated reporter particles labelled with the reference fluorophore AF647SE (magenta). Lysosomes were visualized following the addition of the acidotropic fluorescent dye Lysotracker Red 2 h post-phagocytosis (yellow). Eructophagy was demonstrated by the sudden loss of soluble DQ Green BSA peptide products (green) and Lysotracker Red stain (yellow) from the phagolysosome. Images were captured using an IN Cell Analyzer 2000 between 2 and 4 h post-phagocytosis at 37°C and 5% CO_2. Time 0 represents an eructophagy event. Scale bars denote 10 µm.

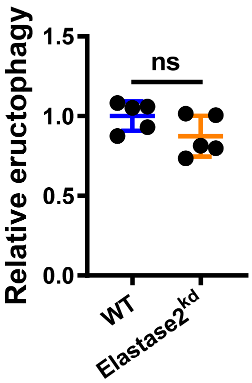

Relative eructophagy
1.5
1.0
0.5
0.0
ns
WT
Elastase2kd

Supplementary Fig. 8. Macrophages derived from conditionally-immortalized myeloid precursors undergo eructophagy and can be genetically manipulated. Knockdown of Elastase 2 by shRNA does not significantly affect eructophagy and is a suitable negative control. Eructophagy was demonstrated through the hydrolysis of membrane impermeable resorufin cellobioside by phagolysosomal cellulase. The membrane-impermeant substrate was added to assay medium 30 min post-phagocytosis of reporter particles. Images were captured on an IN Cell Analyzer 2000 for 2 h, 90 min post-phagocytosis. **n** = 5 per group. Error bars presented as means ± SEM. *P = 0.4 by Student's two-tailed unpaired t-test.

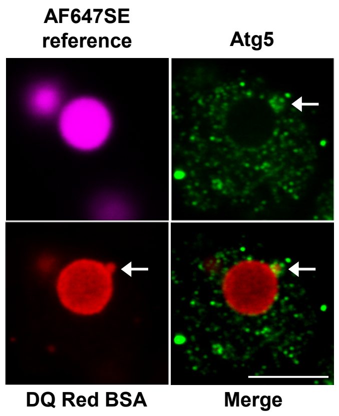
AF647SE
reference
Atg5
DQ Red BSA
Merge

Supplementary Fig. 9. Phagolysosomal blebs are decorated with ATG5. Representative images of fixed BMMØs probed with anti-Atg5 (green) after phagocytosis of experimental particles bearing the self-quenched DQ Red BSA (red) and the reference fluorophore AF647SE (magenta) for 3h. Images were captured using a Leica SP8 Lightning microscope. Scale bars denote 10 µm.

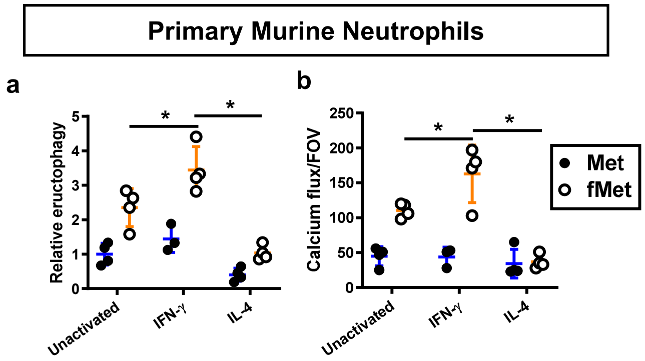

Primary Murine Neutrophils
a
b
Relative eructophagy
Unactivated
IFN-γ
IL-4
Calcium flux/FOV
Met
fMet
*

Supplementary Fig. 10. Primary murine neutrophils are activated by N-formyl-peptide release during eructophagy. (a) Rates of eructophagy as detected by extracellular resorufin maltotriose hydrolysis by phagolysosomal α-amylase reporter particles in WT BMMØs with prior exposure to IFN-γ (100 U/mL, 18 h) or IL-4 (10 ng/mL, 24 h) relative to unstimulated WT BMMØs containing non-formylated (Met) peptides-tethered particles, with co-culture of with murine neutrophils (PMNs). PMNs were added to BMMØs 30 min post-phagocytosis of reporter particles at a ratio of 2:1 (U937/PMN:BMMØ). Rates of eructophagy were recorded between 75 min and 255 min post-phagocytosis. (b) The corresponding incidence of calcium fluxes in co-cultured FPR1-U937 cells per FOV. Calcium fluxes are presented as a numerical average of fluxes detected over ≥ 2 FOV. N=4. Error bars presented as means ± SEM. *P ≤ 0.05 by 2-way ANOVA with Bonferroni comparison of means.

		Eructophagy	Secretory Autophagy
**Major Cell Type**	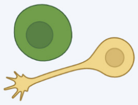	Macrophages	Myeloid cells, epithelial cells, neurons, microglia
**Secretory Vesicle**	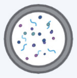	LAMP1+ PL	LAMP1- autophagosome
**Required Proteins**	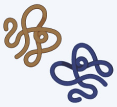	Initial core autophagy proteins (eg. BECN1, ATG5 ATG7, ATG12, PI3K etc.)<br><br>RAB7, VAMP8, STX17, SNAP29<br><br>SEC22B, SNAP23, STX3/4	Initial core autophagy proteins (eg. BECN1, ATG5 ATG7, ATG12, PI3K etc.)<br><br>TRIM cargo receptors<br><br>SEC22B, SNAP23, STX3/4
**LC3 Recruitment**	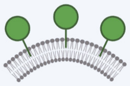	During/Post-secretion	During/Pre-secretion
**Secreted Cargo**	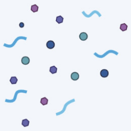	Soluble macromolecules<br>Particulate matter	Soluble macromolecules<br>Aggregate-forming proteins<br>Cytoplasmic organelles<br>Microorganisms
**Fate of Fusion Vesicle**	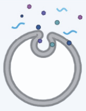	PL-PM fission; PL is translocated into the cell	Autophagosome fuses completely with the PM

Supplementary Fig. 11. Secretory autophagy and eructophagy are distinct processes.

Illustrative depiction of the major similarities and differences of the mechanisms underpinning secretory autophagy and eructophagy.

Protein name	Associated complex	Relative eructophagy
**Elastase 2**	Matrix metalloproteinase 12	0.90 ± 0.06
**Syntaxin 18**	ER Q-SNARE	1.00 ± 0.06
**Syntaxin 1b1**	R-SNARE on synaptic vesicles	1.08 ± 0.01
**Syntaxin 1b2**	R-SNARE on synaptic vesicles	1.15 ± 0.26
**Exoc 8**	Exocyst complex component	0.81 ± 0.077
**SNAPAP**	SNAP associated protein	0.96 ± 0.089
**Syntaxin BP5L**	R-SNARE in neural exocytosis	0.91 ± 0.24
**Rab 28**	Intracellular trafficking	0.82 ± 0.10
**Rab 34**	Phagolysosomal fusion	0.93 ± 0.27
**Syntaxin 3**	Q-SNARE	0.55 ± 0.03
**Syntaxin 4**	Cell membrane Q-SNARE	0.34 ± 0.03
**Vamp 8**	R-SNARE on autophagosomal membrane	0.32 ± 0.02
**Beclin 1**	Autophagy initiation	0.14 ± 0.04
**VPS 41**	HOPS complex	0.36 ± 0.08
**VPS 39**	HOPS complex	0.22 ± 0.03
**VPS 33a**	HOPS complex	0.32 ± 0.04
**Rab 5a**	Early endosome surface	0.62 ± 0.03
**Rab 7**	Late endosome surface	0 ± 0
**Snap 23**	Q-SNARE on plasma membrane	0.28 ± 0.05
**Sec 22b**	R-SNARE on secretory autophagosomes	0.078 ± 0.01
**Syntaxin 17**	Q-SNARE on lysosomal membrane	0.31 ± 0.02

Supplementary Table 1. Specific cellular fusion machinery affects eructophagy. Relative rates of eructophagy in conditionally immortalized precursor-derived macrophages (GB8 macrophages) with specific shRNA-mediated knockdown of fusion machinery. Eructophagy was detected through the hydrolysis of extracellular resorufin cellobioside by phagolysosomal cellulase reporter particles. Data are presented as fold change over WT GB8 macrophages. Fold change > 0.3 was considered a positive screen hit. Knockdown efficiency was determined by qPCR and calculated as relative to 18S and WT control.
