# Supplementary material for: Macrophages disseminate pathogen associated molecular patterns through the direct extracellular release of the soluble content of their phagolysosomes
Source: Nat Commun. 2022 Jun 2;13:3072. doi: 10.1038/s41467-022-30654-4 (PMC9163141; doi:10.1038/s41467-022-30654-4)
Supplement: Supplementary file 3 — Description of Additional Supplementary Files [file 41467_2022_30654_MOESM3_ESM.pdf]

Description of Additional Supplementary Files

Supplementary Movie 1. Transient neutralization of phagolysosomal lumenal pH after phagocytosis of *S. aureus*. Representative sequential images of BMMØs following phagocytosis of paraformaldehyde-fixed *S. aureus* labelled with the pH sensitive fluorophores CFSE (green), pHrodoSE (red) and the reference fluorophore AF647SE (magenta). **Transient neutralization of phagolysosomal lumenal pH** was demonstrated by the transient neutralization of phagolysosomes, as evidenced by concomitant increase in CFSE fluorescence (green) and loss of pHrodoSE fluorescence (red), followed by re-acidification of the phagolysosomal lumen. Images were captured using a Leica SP5 confocal microscope between 45 min and 3 h post-phagocytosis at 37°C. Time is indicated as (hh:mm). Time 00:00 is arbitrary.

Supplementary Movie 2. Transient neutralization of phagolysosomal lumenal pH after phagocytosis of *S. cerevisiae*. Representative sequential images of BMMØs following phagocytosis of paraformaldehyde-fixed *S. cerevisiae* with the pH sensitive fluorophores CFSE (green), pHrodoSE (red) and the reference fluorophore AF647SE (magenta). **Transient neutralization of phagolysosomal lumenal pH** was demonstrated by the transient neutralization of phagolysosomes, as evidenced by concomitant increase in CFSE fluorescence (green) and loss of pHrodoSE fluorescence (red), followed by re-acidification of the phagolysosomal lumen. Images were captured using a Leica SP5 confocal microscope between 45 min and 3 h post-phagocytosis at 37°C. Time is indicated as (hh:mm). Time 00:00 is arbitrary.

Supplementary Movie 3. Sudden loss of soluble green fluorescent peptides with concurrent neutralization of lumenal pH in mature phagolysosomes. Representative sequential images of IFN-γ activated BMMØs following phagocytosis of 3.0 μm reporter particles bearing the quenched DQ Green BSA protease substrate (quenched/green), the pH indicator pHrodoSE (red) and the reference fluorophore AF647SE (magenta). Following complete maturation of the phagosomes to mature phagolysosomes (~90 min), the simultaneous loss of soluble fluorescent peptide products of DQ Green BSA (loss of green signal) and neutralization of the phagolysosomes (loss of red signal) were occasionally observed. Minutes after the loss of lumenal peptides and acidic pH, phagolysosomes were observed to re-acidify (increase in red signal) and resume hydrolysis of the DG Green BSA (increase in green signal). No increase in cytosolic fluorescence was observed following the loss of green fluorescent peptides from the phagolysosome. Images were captured using a Leica SP5 confocal microscope between 1 and 4 h post-phagocytosis at 37°C. Time is indicated as (hh:mm). Time 00:00 is arbitrary.

Supplementary Movie 4. Membrane impermeable fluorescent substrates can transiently enter the phagolysosome and be hydrolysed by particle-restricted enzymes within the phagolysosome. Representative sequential images of BMMØs following phagocytosis of 3.0 μm reporter particles bearing β-glucosidase and the reference fluorophore AF647SE (magenta). Eructophagy was demonstrated by the transient fluorescent signal generated by direct hydrolysis of the membrane impermeable substrate resorufin glucopyranoside (yellow) within the phagolysosome by particle-restricted β-glucosidase. Resorufin glucopyranoside was added to assay medium 30 min post-phagocytosis. Images were captured using a Leica SP5 confocal microscope between 90 min and 4 h post-phagocytosis at 37°C. Time is indicated as (hh:mm). Time 00:00 is arbitrary.

Supplementary Movie 5. Beclin 1 is required for eructophagy. Representative sequential images of BMMØs conditionally-deficient in Beclin 1 (Cre^{+}) and BMMØs derived from Cre negative littermates (Cre^{-}). Eructophagy was detected by hydrolysis of the cell impermeable substrate resorufin maltotriose (yellow) by 3.0 μm reporter particles bearing α-amylase (magenta). Images were captured using a Leica SP5 confocal microscope between 90 min and 3 h post-phagocytosis at 37°C. Time is indicated as (hh:mm). Time 00:00 is arbitrary.

Supplementary Movie 6. LC3-GFP is recruited to the phagolysosomal membrane during eructophagy. Representative sequential images of BMMØs derived from mice expressing LC3-GFP (green) undergoing eructophagy. Eructophagy was detected by hydrolysis of the cell impermeable substrate resorufin maltotriose (yellow) by 3.0 µm reporter particles bearing α-amylase within phagolysosomes between 2 and 4 h following phagocytosis by BMMØs. Images depicting GFP (green) and resorufin (yellow) fluorescent signals are merged. LC3-GFP recruitment to the phagolysosome was observed to be temporally-associated with 88% (± 3.1%) of all eructophagy events observed in these cells. Images were captured using a Leica SP5 confocal microscope between 1-3 h post- phagocytosis at 37°C. Time is indicated as (hh:mm). Time 00:00 is arbitrary.

Supplementary Movie 7. Phagolysosomes retain the ability to fuse with lysosomes immediately following eructophagy. Representative sequential images of BMMØs after phagocytosis of 3.0 µm DQ Green BSA-conjugated reporter particles labelled with the reference fluorophore AF647SE (magenta). Lysosomes were visualized following the addition of the acidotropic fluorescent dye Lysotracker Red 2 h post-phagocytosis (yellow). Eructophagy was demonstrated by the sudden loss of soluble DQ Green BSA peptide products (green) and Lysotracker Red stain (yellow) from the phagolysosome. Images were captured using an IN Cell Analyzer 2000 between 2 and 4 h post-phagocytosis at 37°C and 5% CO2. Time is indicated as (hh:mm). Time 00:00 is arbitrary.

Supplementary Movie 8. Blebbing is associated with the phagolysosomal membrane immediately prior to eructophagic events. Representative sequential images of BMMØs following the phagocytosis of experimental particles bearing DQ Green BSA (green), pHrodoSE (red) and AF647SE (magenta). Phagolysosomal morphology can be visualized in the green channel as the soluble DQ Green BSA peptide products are not particle-restricted and hence occupy the entire phagolysosomal lumen. The green signal within the phagolysosome was purposely saturated to visualize the smaller, less intense bleb. A single ~0.5 µm diameter bleb of the phagolysosomal membrane is commonly observed before an eructophagic event (as demonstrated by the sudden loss of soluble DQ Green BSA peptide products to the extracellular space). Images were captured using a Leica SP5 confocal microscope between 1-3 h post-phagocytosis at 37°C. Time is indicated as (hh:mm). Time 00:00 is arbitrary.

Supplementary Movie 9. ATG5 is found on phagolysosomal blebs. Representative 3D reconstruction of a single phagolysosome undergoing blebbing. BMMØs were formalin fixed following the phagocytosis of experimental particles bearing DQ Red BSA (red), and AF647SE (magenta). Presence of ATG5 on the bleb was examined using an anti-ATG5 antibody (green). Blebs are seen in the red channel as the soluble peptide product from the phagolysosome occupies the entire phagolysosome. Images were captured using a Leica SP8 Lightning microscope. Scale bar represents 2µm.

Supplementary Movie 10. Formylated peptides released during eructophagy activate vicinal cells. Following phagocytosis of reporter particles with tethered formylated (fMet) or non-formylated (Met) peptides, BMMØs were co-cultured with FPR1-expressing U937 cells loaded with the calcium-sensor Fluo-4 AM. Sequential confocal images demonstrating temporal and spatial relationships between an eructophagic event (yellow) in BMMØs (as detected by cellulase-reporter beads [magenta] with tethered fMet peptides) and cytosolic calcium fluxes (green) in vicinal U937 cells. Images were captured using an IN Cell Analyzer 2000 between 1-2 h post-phagocytosis at 37°C and 5% CO2. Brightfield images are shown at two different focal plains to show adherent BMMØs (top left panel) and suspension U937 (top middle panel) cells. Time is indicated as (hh:mm). Time 00:00:00 is arbitrary.
