# Supplementary material for: Macrophages disseminate pathogen associated molecular patterns through the direct extracellular release of the soluble content of their phagolysosomes
Source: Nat Commun. 2022 Jun 2;13:3072. doi: 10.1038/s41467-022-30654-4 (PMC9163141; doi:10.1038/s41467-022-30654-4)
Supplement: Supplementary file 14 — Reporting Summary [file 41467_2022_30654_MOESM14_ESM.pdf]

nature portfolio

Corresponding author(s): Yates, R.M.

Last updated by author(s): Jan 20, 2022

Reporting Summary

Nature Portfolio wishes to improve the reproducibility of the work that we publish. This form provides structure for consistency and transparency in reporting. For further information on Nature Portfolio policies, see our Editorial Policies and the Editorial Policy Checklist.

Statistics

For all statistical analyses, confirm that the following items are present in the figure legend, table legend, main text, or Methods section.

n/a	Confirmed	
☐	☒	The exact sample size (*n*) for each experimental group/condition, given as a discrete number and unit of measurement
☐	☒	A statement on whether measurements were taken from distinct samples or whether the same sample was measured repeatedly
☐	☒	The statistical test(s) used AND whether they are one- or two-sided<br>*Only common tests should be described solely by name; describe more complex techniques in the Methods section.*
☐	☒	A description of all covariates tested
☐	☒	A description of any assumptions or corrections, such as tests of normality and adjustment for multiple comparisons
☐	☒	A full description of the statistical parameters including central tendency (e.g. means) or other basic estimates (e.g. regression coefficient) AND variation (e.g. standard deviation) or associated estimates of uncertainty (e.g. confidence intervals)
☐	☒	For null hypothesis testing, the test statistic (e.g. *F*, *t*, *r*) with confidence intervals, effect sizes, degrees of freedom and *P* value noted<br>*Give P values as exact values whenever suitable.*
☒	☐	For Bayesian analysis, information on the choice of priors and Markov chain Monte Carlo settings
☒	☐	For hierarchical and complex designs, identification of the appropriate level for tests and full reporting of outcomes
☒	☐	Estimates of effect sizes (e.g. Cohen's *d*, Pearson's *r*), indicating how they were calculated

Our web collection on statistics for biologists contains articles on many of the points above.

Software and code

Policy information about availability of computer code

Data collection	IN Cell 2000 Analyzer v. 5.2, LAS Application Suite for Leica SP5, LAS Application Suite for Leica SP8 with Lightning.
Data analysis	IN Cell Investigator 1.8.3 software was used to analyze data in conjunction with Spotfire DecisionSite® Client software (TIBCO Software Incorporated) and a custom designed analysis app based on R. Representative images and videos were processed using Image J. GraphPad Prism® versions 5.0 and 8.0 were used for statistical analysis.

For manuscripts utilizing custom algorithms or software that are central to the research but not yet described in published literature, software must be made available to editors and reviewers. We strongly encourage code deposition in a community repository (e.g. GitHub). See the Nature Portfolio guidelines for submitting code & software for further information.

Data

Policy information about availability of data

All manuscripts must include a data availability statement. This statement should provide the following information, where applicable:

- Accession codes, unique identifiers, or web links for publicly available datasets
- A description of any restrictions on data availability
- For clinical datasets or third party data, please ensure that the statement adheres to our policy

The datasets generated during and/or analysed during the current study are available from the corresponding author (rmyates@ucalgary.ca) on reasonable request. We have also provided a source data file for all figures. As our data is primarily image based, currently it is not possible to make it accessible to the public.

Field-specific reporting

Please select the one below that is the best fit for your research. If you are not sure, read the appropriate sections before making your selection.

- [x] Life sciences
- [ ] Behavioural & social sciences
- [ ] Ecological, evolutionary & environmental sciences

For a reference copy of the document with all sections, see nature.com/documents/nr-reporting-summary-flat.pdf

Life sciences study design

All studies must disclose on these points even when the disclosure is negative.

Sample size	For experiments involving animals, n=3 was chosen as the minimal sample size. In our previous experiments, n=3 provided sufficient representation of the population of these inbred mice. For experiments involving cell lines, n=3 was chosen as the minimal well replicate number for at least 3 independent experiments. With these sample sizes we are confident that our data is a real finding and not an experimental error. No statistical methods were used to determine sample size for experiments.
Data exclusions	Data was excluded for macrophages derived from conditional knockout animals (LysM Cre) or GB8 macrophages where we saw no excision of the target protein or knockdown of the gene target.
Replication	Experiments were successfully replicated at least twice for each animal studied. Well replicates for image analysis were at least 2 wells. For plate assays, at least three well replicates were imaged for at least 3 independent experiments. For quantification of blebs, 5 randomly selected fields of view from 3 independent replicate slides were imaged.
Randomization	Animals were age matched to WT control mice and were randomly selected from mice between 8-12 weeks old. Fields of view from well or slide replicates were randomly selected for analysis and/or quantification.
Blinding	Fully blinded studies were not conducted as the investigator who extracted bone marrow from the mice, derived the macrophages and conducted the assay. The same investigator conducted data analysis. For image analysis, a custom designed app based on R was used to analyze the data generated by the IN Cell Investigator software. The use of the app removed bias from the analysis.

Reporting for specific materials, systems and methods

We require information from authors about some types of materials, experimental systems and methods used in many studies. Here, indicate whether each material, system or method listed is relevant to your study. If you are not sure if a list item applies to your research, read the appropriate section before selecting a response.

Materials & experimental systems

n/a	Involved in the study	
[ ]	[x]	Antibodies
[ ]	[x]	Eukaryotic cell lines
[x]	[ ]	Palaeontology and archaeology
[ ]	[x]	Animals and other organisms
[ ]	[x]	Human research participants
[x]	[ ]	Clinical data
[x]	[ ]	Dual use research of concern

Methods

n/a	Involved in the study	
[x]	[ ]	ChIP-seq
[x]	[ ]	Flow cytometry
[x]	[ ]	MRI-based neuroimaging

Antibodies

Antibodies used	A CD11b positive selection kit was used to enrich for murine splenic macrophages from Stem Cell Technologies #18970. A CD14+ kit was used to enrich for human peripheral blood monocytes from Miltenyi Tech #130-050-20. Rabbit anti-Atg5 (Proteintech, 10181-2-AP). Goat anti-rabbit secondary antibody conjugated to Alexa Fluor 488 (Thermo Fisher Scientific).
Validation	The CD11b+ kit in Cd11b+ cell isolation from murine spleens was validated by the manufacturer's website. CD14+ kit cites the following on manufacturer's website: Hanley, P. J. et al. (2004) Extracellular ATP induces oscillations of intracellular Ca2+ and membrane potential and promotes transcription of IL-6 in macrophages. Proc. Natl. Acad. Sci. U.S.A. 101: 9479-9484. For their use in immunofluorescence, anti-Atg5 was validated on its manufacturer website and by previous studies.

Eukaryotic cell lines

Policy information about cell lines

Cell line source(s)	U937 - human histiocytic lymphoma patient, GB8 cells - derived from murine bone marrow in house, HEK Blue TLR9 and HEK Blue Null - human embryonic kidney cells. HEK Blue TLR9 (hkb-htlr9) and HEK Blue Null cells (hkb-null1) were purchased from

InvivoGen. U937 cells were a gift from Dr. Eric Prossnitz, University of New Mexico.

Authentication	U937 cells over-expressing FPR1 receptor were authenticated as being responsive to the FPR1 agonist, formylated-methionine through confocal imaging of calcium flux in live cells. HEK Blue TLR9 cells were authenticated as being responsive to the TLR9 agonist, ODN DNA by quantitative PCR. Gene knockdowns in GB8 cell lines were validated with quantitative PCR during assays.
Mycoplasma contamination	All cells tested negative for mycoplasma contamination by PCR analysis prior to use.
Commonly misidentified lines (See ICLAC register)	*Name any commonly misidentified cell lines used in the study and provide a rationale for their use.*

Animals and other organisms

Policy information about studies involving animals; ARRIVE guidelines recommended for reporting animal research

Laboratory animals	All mice were used between 8-12 weeks of age and gender was not a factor in these studies; both male and female mice were used. The following strains were used: C57BL/6J (WT), TSC1fl/fl, B6.129S6-Cybb-/- (Cybb-/-) PI3Kγ-/-, MyD88-/-, B6.129P2-Lyz2 mice (lysMCre), Beclin 1fl/fl and MyD88/TRIF-/-, ATG5fl/fl, ATG7fl/fl, Rubicon-/- and LC3-GFP.<br><br>The following strains were bred at the University of Calgary under standard pathogen-free conditions in individually ventilated cages at room temperature (19-23ºC) with 45-55% humidity and 12-hour light/dark cycle: C57BL/6J (WT), TSC1fl/fl , B6.129S6-Cybb-/- (Cybb-/-), MyD88-/-, PI3Kγ-/- , B6.129P2-Lyz2 mice (lysM-Cre), Beclin 1fl/fl and MyD88/TRIF-/-. With the exception of Beclin 1fl/fl and MyD88/TRIF-/-, all miec were purchased from The Jackson Laboratory. Beclin 1fl/fl mice were provided by Dr. Edmund Rucker (University of Kentucky, KY, USA) and MyD88/TRIF-/- mice were provided by Dr. Paul Kubes (University of Calgary, Canada).<br><br>The following strains were bred at Washington University, St Louis under standard pathogen-free conditions: ATG5fl/fl, ATG16Lfl/fl, ATG7fl/fl, Rubicon-/-and LC3-GFP.
Wild animals	This study did not involve wild animals
Field-collected samples	Study does not contain field collected samples
Ethics oversight	Mouse ethics approval came from the Animal Care and Use Committee at the University of Calgary or Washington University, St. Louis, designed in accordance to the Canadian Council of Animal Care (where applicable). Human blood samples were used as approved by the Conjoint Health Research Ethics Board at the University of Calgary.

Note that full information on the approval of the study protocol must also be provided in the manuscript.

Human research participants

Policy information about studies involving human research participants

Population characteristics	All volunteers were healthy with no none underlying conditions on the day of sampling.
Recruitment	Healthy human volunteers were recruited to give blood from a database of previous and current volunteers. Blood was extracted by a research phlebotomist. Main recruitment factors considered were availability of the volunteer and their current health status.
Ethics oversight	Approved by the Conjoint Health Research Ethics Board at the University of Calgary

Note that full information on the approval of the study protocol must also be provided in the manuscript.
